# See-Through Type 3D Head-Mounted Display–Based Surgical Microscope System for Microsurgery: A Feasibility Study

**DOI:** 10.2196/11251

**Published:** 2019-03-07

**Authors:** Cheol-Hwan Kim, Seon-Young Ryu, Ji-Young Yoon, Hyoung-Kwon Lee, Nak-Gu Choi, Il-Ho Park, Hae-Young Choi

**Affiliations:** 1 Institute of Advanced Convergence Technology Kyungpook National University Daegu Republic of Korea; 2 Department of Creative IT Engineering Pohang University of Science and Technology Pohang Republic of Korea; 3 Medical Device Development Center Daegu-Gyeongbuk Medical Innovation Foundation Daegu Republic of Korea; 4 Research Laboratory Green Optics CO Cheongju Republic of Korea; 5 Research Lab Hulust CO Wonju Republic of Korea; 6 Department of Otorhinolaryngology Korea University Medical College Seoul Republic of Korea

**Keywords:** 3D imaging, head-mounted display, microsurgery, surgical microscope

## Abstract

**Background:**

The surgical microscope is used primarily for microsurgeries, which are more complicated than other surgical procedures and require delicate tasks for a long time. Therefore, during these surgical procedures, surgeons experience back and neck pain. To solve this problem, new technology, such as wearable displays, is required to help surgeons maintain comfortable postures and enjoy advanced functionality during microsurgery.

**Objective:**

The objective of this study was to develop a surgical microscope system that would work with wearable devices. It would include a head-mounted display (HMD) that can offer 3D surgical images and allow a flexible and comfortable posture instead of fixed eyepieces of surgical microscope and can also provide peripheral visual field with its optical see-through function.

**Methods:**

We designed and fabricated a surgical microscope system that incorporates a see-through type 3D HMD, and we developed an image processing software to provide better image quality. The usability of the proposed system was confirmed with preclinical examination. Seven ENT (ear, nose, and throat) surgical specialists and 8 residents performed a mock surgery—axillary lymph node dissection on a rat. They alternated between looking through the eyepieces of the surgical microscope and viewing a 3D HMD screen connected to the surgical microscope. We examined the success of the surgery and asked the specialists and residents to grade eye fatigue on a scale of 0 (none) to 6 (severe) and posture discomfort on a scale of 1 (none) to 5 (severe). Furthermore, a statistical comparison was performed using 2-tailed paired t test, and *P*=.00083 was considered significant.

**Results:**

Although 3D HMD case showed a slightly better result regarding visual discomfort (*P*=.097), the average eye fatigue was not significantly different between eyepiece and 3D HMD cases (*P*=.79). However, the average posture discomfort, especially in neck and shoulder, was lower with 3D HMD display use than with eyepiece use (*P*=.00083).

**Conclusions:**

We developed a see-through type 3D HMD–based surgical microscope system and showed through preclinical testing that the system could help reduce posture discomfort. The proposed system, with its advanced functions, could be a promising new technique for microsurgery.

## Introduction

The surgical microscope is mainly used for microsurgeries, such as in neurosurgical application, ENT (ear, nose, and throat) surgeries, ophthalmic surgeries, plastic and reconstructive surgeries, and dental treatments. Microsurgery requires accurate and stable task performance with small and delicate instruments, and surgeons spend long sessions with their eyes on the eyepieces of the microscope. Therefore, due to their awkward static postures during operations, surgeons experience back and neck pain, musculoskeletal fatigue, and injuries [1-4]. In a national cross-sectional survey of 325 ENT consultants by Babar-Craig et al [2], 72% reported having experienced either back or neck pain or both, with most reports coming from otologists, relating their symptoms to lengthy microscopic work and prolonged sitting. Overall, 53% of respondents attributed their symptoms directly to previously performed ENT surgeries. Other predisposing factors include static postures and bending during endoscopic procedures, similar to their general surgical colleagues [2]. A survey of 5 different specialists in the United Kingdom—general surgery, otorhinolaryngology, plastic surgery, orthopedics, and trauma surgery and neurosurgery—indicated that the back and neck were the most common areas of pain. Posture was the most common cause of pain (46%), followed by the use of microscopes or surgical instruments (21% each) and surgical loupes or head-mounted lights (11%) [3]. However, many surgeons pay little attention to their health, and their work-related illness has been reported to be above average compared with that of professionals from other industries [1]. Therefore, new technologies should be adopted in the workplace to reduce posture-related discomfort, provide better visualization during surgical procedures, and help with pain management.

Heads-up microscopy using a high-definition (HD) 3D monitor has been proposed as an ergonomic alternative. This system transmits the surgical microscopic images to a 3D monitor for the surgeon and assistants to view in more comfortable positions. In addition, it allows the surgeon to recognize the surgical environment easily [5]. However, in heads-up microscopy, a surgeon’s head and eyes are directed toward a monitor, which causes an eye-hand coordination mismatch and requires an uncomfortable posture for surgeons. Head-mounted displays (HMDs) have been adopted to solve the abovementioned drawbacks in the case of endoscopy and laparoscopy, which use the same kinds of 3D monitors [6-13]. The use of HMD helps eliminate these problems by delivering optical information directly to the surgeon’s eyes, independent of the head and body position and the position of the sources of images. Therefore, HMDs can relieve back and neck strain and improve technical proficiency. In addition, 3D functions of the HMD-based laparoscopic system and endoscopic surgery system provide benefits such as improved operation times, minimized complications, shortened learning curves, and greater surgeon comfort [14,15].

Despite the several advantages of the HMD, the associated disadvantages still limit its use in surgery. Many of the proposed HMDs have inadequate resolution, and they are bulky, cave-like, and heavy [6-10]. Although a higher-resolution HMD has recently been proposed for endoscopy, it has a fully opaque and closed, nonsee-through configuration [11-13]. Because this type of HMD can only deliver virtual information to the eye, it is difficult to obtain a direct physical view and to observe the surgical environment in the operating room. Therefore, in this study, we propose a surgical microscope system based on a high-resolution 3D HMD with a see-through configuration that enables optical superposition of digital information onto the physical view. Using the proposed system, 3D surgical images can be observed with more comfortable postures, and a peripheral view of the surgical field can also be easily accessed during microsurgery via the see-through configuration. We designed and fabricated a stereoscopic surgical microscope and see-through 3D HMD and developed image processing software. The feasibility of the proposed HMD-based surgical microscope system was evaluated with preclinical examination, and the results were compared with the results obtained with the conventional eyepieces of the system.

## Methods

### Surgical Microscope System Using Head-Mounted Display

**Figure 1:**
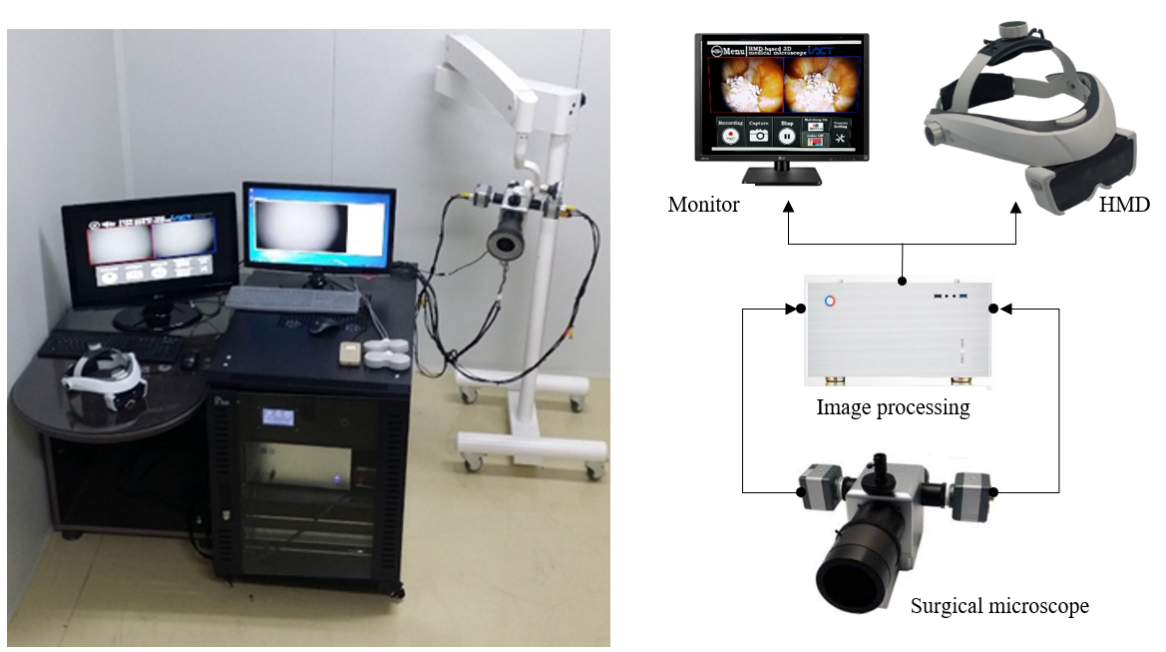
Left: surgical microscope system based on see-through type 3D head-mounted display (HMD); right: surgical images measured using 2 charge-coupled device cameras are displayed on a monitor in two dimensions and displayed on HMD in 3D after the image processing procedure.

We implemented a surgical microscope system incorporating a see-through type 3D HMD and image processing computer, as shown in Figure 1 (image on the left). A surgical microscope was developed that comprises a binocular headpiece with adjustable eyepieces and 2 charge-coupled device (CCD) cameras, optical see-through type 3D HMD mounted on a headband, and image processing software. The measured surgical images are directly observed through eyepieces, and the measured surgical images from the 2 CCD cameras on the surgical microscope are sent to an image processing computer for better image quality and for 3D image reconstruction in real time, as described in Figure 1 (image on the right). The processed images are displayed on a monitor and the proposed 3D HMD. Furthermore, two-dimensional (2D) side-by-side images are displayed on the monitor, and 3D surgical images are displayed by the HMD.

### Stereoscopic Microscope

**Figure 2:**
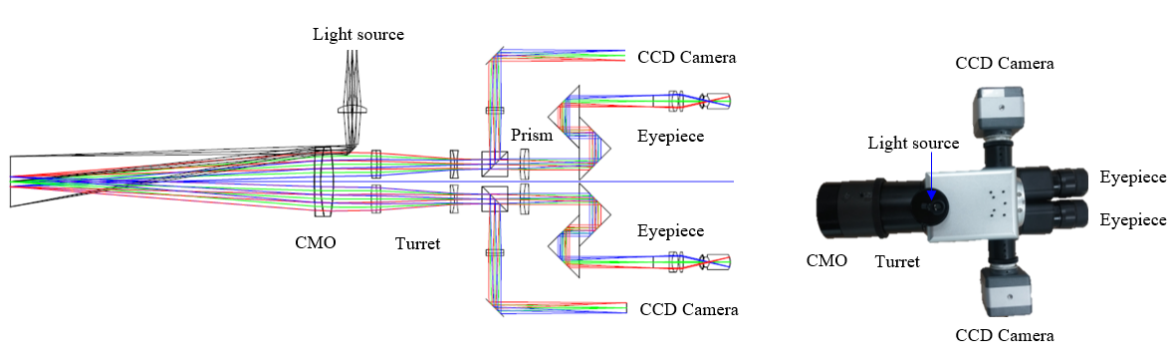
Left: optical design of the stereoscopic microscope; right: picture of the fabricated stereoscopic microscope. CCTV: closed-circuit television; CCD: charge-coupled device; CMO: common main objective.

We designed and fabricated a stereoscopic microscope with 2 CCD cameras for use as a surgical microscope. The device used a binocular design with a single common main objective, as described in Figure 2. The microscope comprised a light source, 2 CCD cameras, and optical components: one common main objective, a pair of turrets, a prism, an eyepiece, and a closed-circuit television (CCTV) lens. A white light emitting diode light source (NET-260SL, Mega Medical Co Ltd) was attached to the illumination port of the microscope. The continuous zooming turret, having a focusing component, a zooming component, and a compensating component was adopted, and it was connected to a DC motor and foot switch. A varifocal lens with a motor drive was used for autofocusing. The step control was 0.18 degrees, and the autofocusing precision was 1.2 µm. The magnification ratio was 1× to 4×, and the object distance was 200-400 mm. The CCTV lens was connected to the turret lens, and it delivered images to the CCD cameras. HD CCD cameras (HDC-SD041BS, WatchCam Co Ltd) were attached next to the CCTV lenses. The camera had a 1/3-inch complementary metal-oxide-semiconductor sensor, and the total pixels were 2010 (H)×1108 (V). The video output was set to 1280×720, which was the same as the pixel size of the microdisplay for the HMD. The measured resolution of the microscope was 79 line pairs (lp) at 200-mm distance and 105 lp at 400-mm distance. The microscope was implemented with water resistance of IPX4. The specifications of the optical system of the proposed stereoscopic microscope are listed in Table 1.

**Table 1:** Specification of the optical system of the proposed stereoscopic microscope.

Feature	Specification
Resolution	79 line pairs at 200 mm
Charge-coupled device resolution	1280×720
Magnification	Continuous zoom (1:4)
Object distance	200-400 mm
Autofocusing resolution	1.2 µm
Waterproof	IPX4
Display	Eyepieces, monitor, head-mounted display

### See-Through Type 3D Head-Mounted Display

**Figure 3:**
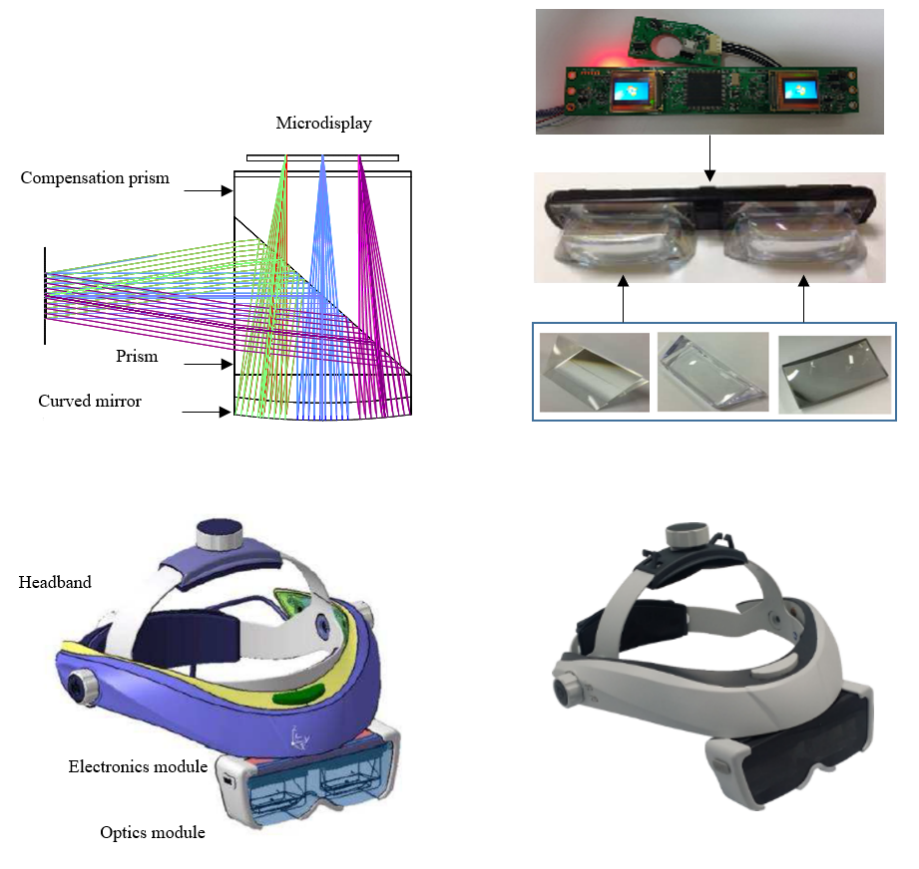
Top left: optical design of see-through type 3D head-mounted display (HMD); top right: integration of the control board and the optical module; bottom left: headband design; bottom right: fabricated see-through type 3D HMD.

A see-through type 3D HMD was designed and fabricated for obtaining not only the 3D surgical image but also a direct physical sight. The binocular design has 2 separate displays with 2 input channels, 1 for each eye, which makes it possible to observe stereoscopic images. Optics designing software (ZEMAX LLC, Optic Studio) was used to create an optical design using 2 prisms and a curved mirror. As shown in Figure 3 (top left image), the image from the microdisplay was enlarged and reflected by the curved mirror and then transferred to the eye after being reflected on the surface of the prism. Using a compensation prism, the sight from outside can also be seen at eye position without distortion. A polarizing film was attached on the prism surface, and a 1/4 λ wave plate was placed between the prism and the curved mirror to minimize optical power loss. The polarizing film can only transmit p-waves and reflect s-waves, and the efficiencies of both are over 80%. Therefore, although the nonpolarized input beam is reduced to half of its original amount by the polarizing film, 40% of p-waves are transmitted. The transmitted p-waves are rotated to s-waves by passing through the 1/4 wave plate twice. Finally, over 34% of the input beam is delivered to the eye. The curved mirrors are fabricated of a glass material (N-BK7). To reduce the weight of optical module, polymethyl methacrylate was used in the fabrication of the optical prisms that are most bulky part in the optical module, and the outside of the effective optical path in optical prisms were cut, as shown in Figure 3 (top right image). Optical components were attached using optical adhesive, and 2 optical modules were placed at the designed positions in relation to the microdisplay.

A 0.61-inch organic light emitting diode display (MDP02BCWF, MICROOLED) with pixel sizes of 1280×1024 was used as a high-resolution microdisplay. The output pixels were set to 1280×720 pixels. The microdisplay driving board with a high-definition multimedia interface (HDMI) and USB power port was custom fabricated. The electric control board was assembled with the optical module, as shown in Figure 3 (top right). The integrated HMD module was mounted on a headband, and the HDMI cable and USB power cable were housed at the rear of the headband for comfortable weight balance. Figure 3 (bottom images) shows the design of the HMD and the fabricated item itself. The headband is designed to adjust in size to fit any user’s head. The height and angle of the HMD module were made to be adjustable to match a user’s eye. The performance of HMD was measured using a home-constructed measurement system and approved target. The measured field of view was 41 degrees in the diagonal direction, and the distortion was 2.4%. The eye relief was 15 mm, and the horizontal and vertical dimensions of the eye box were 10 mm and 4 mm, respectively. The measured weight of the HMD, without the headband, was about 92 g. To deliver the surgical image dominantly, a polarization window with <10% transparency was applied in front of the HMD. Therefore, we can observe the 3D surgical image as the primary image in the field of view and can also acquire a peripheral view outside the effective area. The specifications of the HMD are listed in Table 2.

**Table 2:** Specification of the head-mounted display.

Feature	Specification
Resolution	High definition (1280×720)
Display	Organic light emitting diode
Type	See-through (2D or 3D)
Field of view	41 degrees (diagonal)
Eye Relief	15 mm
Eye box	10 mm×4 mm (H×V)
Distortion	2.4%
Interface	High-definition multimedia interface
Weight	92 g (without headband)

**Figure 4:**
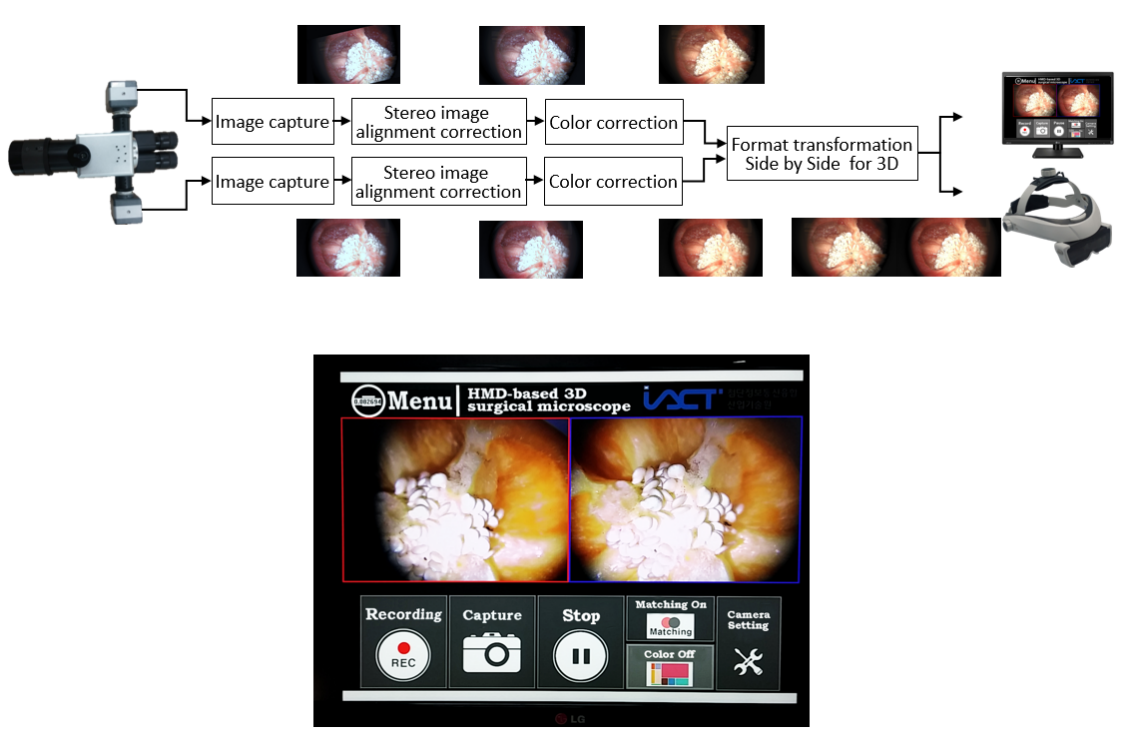
Top: image processing flow; bottom: system control and image processing software.

### Image Processing Software

The key roles of the image processing software are to improve the image quality and transform the format for 3D HMD. In general, the side-by-side 3D image is made from half-size left and right images reduced in the horizontal dimension, but we used full-size left and right images to achieve a better image quality for medical applications. As shown in Figure 4 (top image), the captured 1280×720 left and right microscope images are transformed into a 2560×720 side-by-side image for HMD through two main image processing procedures: stereo image alignment correction and color correction. The stereo image alignment correction function is intended to restore left and right image alignment error caused by optical misalignment of the microscope. In the stereo image alignment correction process, the left and right images are converted by homography matrix. The matched feature point pairs extracted using by Speeded-Up Robust Features algorithm in the left and right images are used to calculate the homography matrix [16,17]. After correcting the stereo image alignment, the vertical error of the matched feature points was 0%, the rotational error was 1.1°, and the zoom size error was 2%. The color correction function is intended to repair the color distortion caused by the characteristics of the camera image sensor and, therefore, to make the color of the image equal to the color of the real object. We used the colorimetric matching method to correct color distortion [18,19]. In this method, the characteristics of the camera sensor are modeled from the XYZ values of the original object measured using the color meter device and the RGB (red, green, and blue) values of the camera image. After finding the matrix of the camera model, the invert matrix of the camera matrix is used to convert the camera image to its original color. We used the 140-color ColorChecker to find the matrix of the camera model; after correcting the color, the average CIELAB color difference between the measured color and corrected color for the 140 patches was 4.35.

In addition, we developed the graphical user interface for user convenience functions, such as still shot, recording, play, and pause, as shown in Figure 4 (bottom image). Furthermore, camera settings such as brightness, contrast, and sharpness can be directly controlled, which reduces software load for image processing.

## Results

### Head-Mounted Display–Based Surgical Microscope System Performance

**Figure 5:**
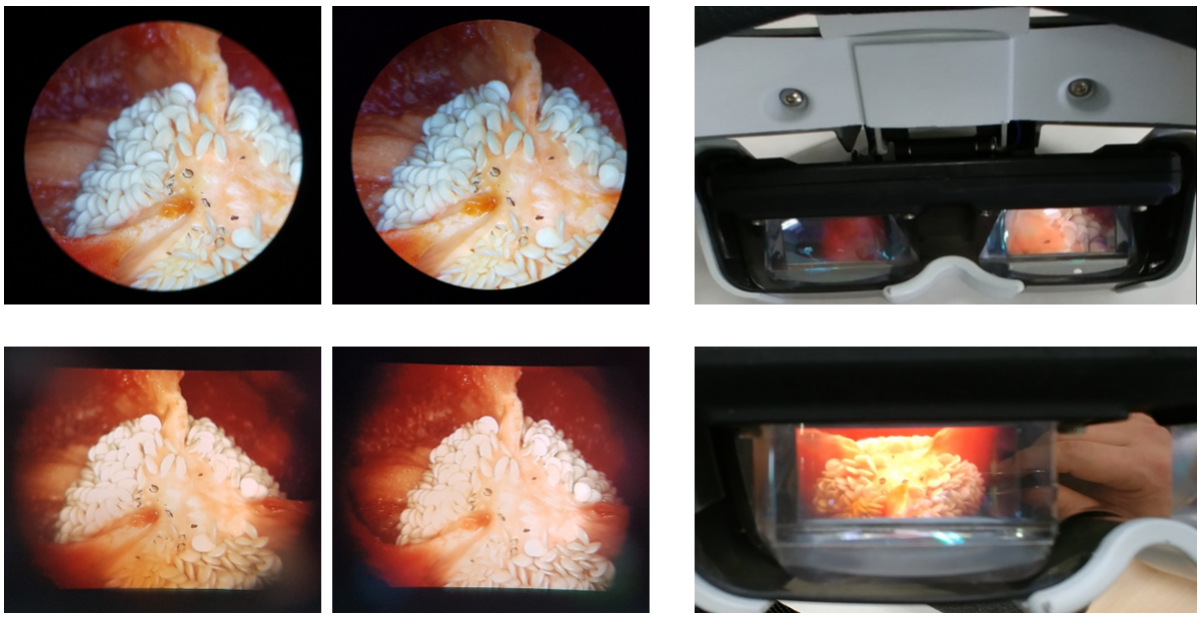
Top left: measured left and right images using eyepieces; bottom left: measured left and right images using head-mounted display (HMD); top and bottom right: a demonstration of the see-through type 3D HMD.

The performance of the proposed system was evaluated by measuring test samples of *Capsicum annuum*. Figure 5 represents left and right images directly captured at the eyepieces (top left) and HMD (bottom left) at eye position. By those slightly different angled images, we can observe 3D images through the eyepieces and the HMD. Different brightness and fields of view were observed because of the different optics between the eyepieces and the CCD camera. Brighter images were observed through the eyepieces and better fields of view were observed via the HMD, as shown in Figure 5 (top and bottom left images, respectively). The camera setting and image processing procedure were optimized to acquire microscope-like 3D reconstruction images from the HMD that were more comfortable to eyes. Through the HMD, we can mainly observe the measured object and can also recognize environmental situation from peripheral view due to its optical see-through design, as shown in Figure 5 (top and bottom right images).

### Preclinical Study

**Figure 6:**
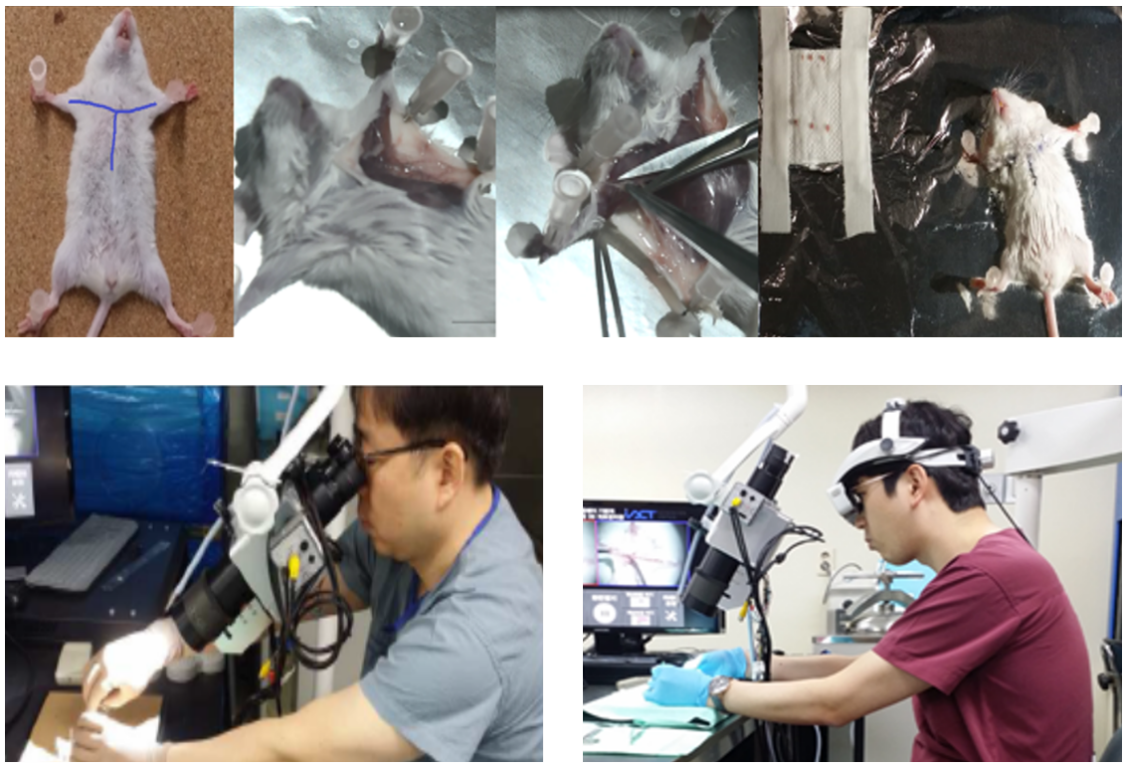
Top: preclinical test of axillary lymph node dissection in white mice; bottom left: preclinical test using eyepieces; bottom right: preclinical test using see-through type 3D head-mounted display.

We tested the feasibility of the see-through type 3D HMD–based surgical microscope system through preclinical trial surgery. The preclinical experiment was conducted according to the protocol of the Korea University Medical Center. Male mice aged 8 weeks were used. Experimental methods involving animals were approved by the Institutional Animal Care and Use Committee of Korea University. Mice were anesthetized with 2.5% isoflurane. The surgical sites were aseptically prepared and draped to provide a sterile field. The axillary lymph nodes were exposed after dissection of the skin and fascia (Figure 6, top image); 3 lymph nodes were collected from each site. The collected lymph nodes were checked and confirmed by another surgeon. A nonabsorbable suture was used for skin closure. Suture removal was performed 7 days later under isoflurane anesthesia as necessary. Mice were checked-up once a day until suture removal, then once every week for 1 month.

We used the proposed surgical microscope with and without the see-through type 3D HMD for comparison, as shown in Figure 6 (bottom images). The experiment was a preclinical test of axillary lymph node dissection in white mice. Seven ENT surgical specialists and 8 residents participated in the test, all of whom were experts in microscopic procedures. The evaluation items were the success of the operation, the eye fatigue test, and the postural discomfort test.

First, the success of the operation was evaluated with regard to 4 different categories, as shown in Table 3. The failure criteria for the preclinical trial surgery were as follows: (1) if the operation time exceeds 1 hour; (2) if the tester cannot collect >6 lymph nodes; and (3) if white mice have been bleeding or are unable to survive.

As shown in Table 3, the experimental results demonstrated that 6 trials of surgeries using the proposed 3D HMD–based surgical microscope were successfully performed without bleeding in mice. The average operation time was similar with eyepieces and with the 3D HMD.

**Table 3:** Comparison of preclinical experiment results obtained using head-mounted display (HMD) and eyepieces.

Category and device		Result	Success or Failure	Experiment success criteria
**Average operation time**				
	HMD	21 minutes 41 seconds	Success	1 hour
	Eyepieces	22 minutes 42 seconds	Success	
**Number of lymph nodes sampled**				
	HMD	6	Success	6
	Eyepieces	6	Success	
**Bleeding**				
	HMD	No	Success	No
	Eyepieces	No	Success	
**Survival**				
	HMD	Survival	Success	Survival
	Eyepieces	Survival	Success	

The eye fatigue test measured subjective fatigue from 0 to 7 points for tired eyes, sore or aching eyes, irritated eyes, dry eyes, eyestrain, hot or burning eyes, blurred vision, difficulty focusing, and visual discomfort. In addition, headache, dizziness, nausea, and decreased concentration were evaluated. The eye fatigue test was evaluated before and after the preclinical trial surgery tests.

**Figure 7:**
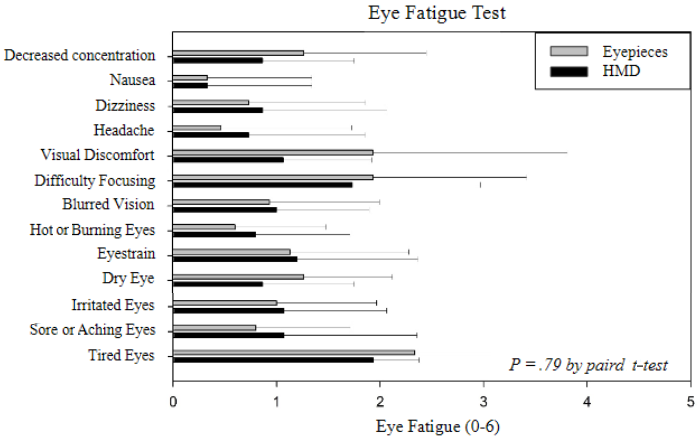
Results of eye fatigue test. HMD: head-mounted display.

In the preclinical test using the eyepieces of the surgical microscope, the eye fatigue scores were in the order of tired eyes (2.3), difficulty focusing (1.9), and vision discomfort (1.9). Using the proposed 3D HMD technology, the results were in the order of tired eyes (1.9), difficulty focusing (1.7), and eyestrain (1.2). The 2-tailed paired *t* test of tired eyes showed no significant difference between the use of eyepieces and the use of HMD (*P*=.79). However, in the case of visual discomfort, the *P* value was .097 (Figure 7), which means that the HMD has the potential to help reduce visual discomfort.

**Figure 8:**
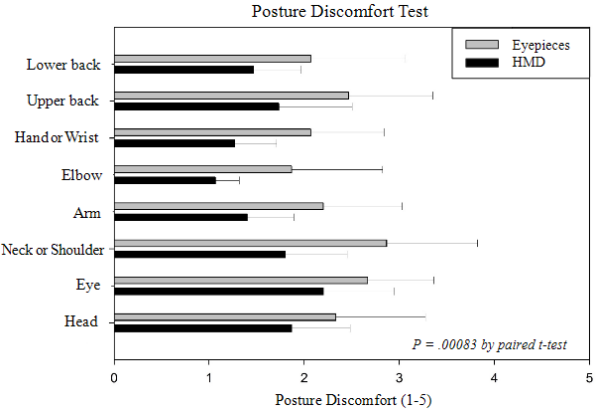
Results of posture discomfort test. HMD: head-mounted display.

The posture discomfort test measured discomfort with a rating of 1 to 5 points for discomfort associated with the lower back, upper back, hand or wrist, elbow, arm, neck or shoulder, eye, and head after the preclinical test. After the preclinical test using the eyepieces of the surgical microscope, the posture discomfort scores were in the order of neck or shoulder (2.9), eyes (2.7), and upper back (2.5). Using the HMD-based surgical microscope, the scores were in the order of eyes (2.2), head (1.9), and neck or shoulder (1.8). The mean of the posture discomfort score with and without using the HMD was 12.5 and 18.5, respectively. The results of the paired *t* test of posture discomfort scores showed a significant difference between the use of the eyepieces and the use of the HMD (*P*=.00083; Figure 8); these results confirmed that the proposed see-through type 3D HMD can reduce posture discomfort, especially regarding neck and shoulder pain, during surgical procedures.

**Figure 9:**
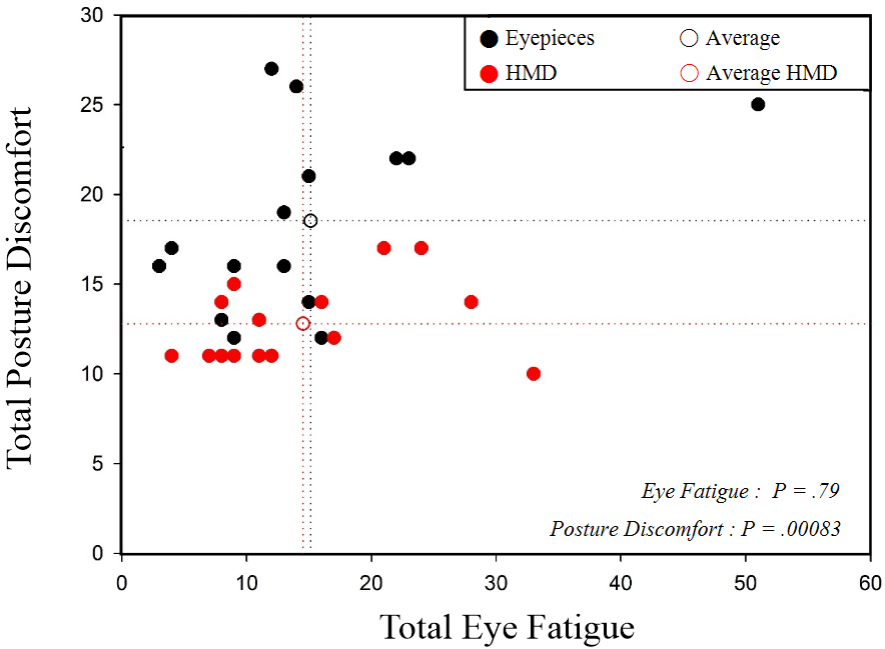
Comparison of eye fatigue and posture discomfort scores for head-mounted display (HMD) and eyepieces.

We analyzed the total scores for eye fatigue and posture discomfort. Using the eyepieces, the eye fatigue and posture discomfort scores were distributed from 3 to 51 points (average 15.1) and 12 to 27 points (average 18.5), respectively. Using the 3D HMD, the eye fatigue and posture discomfort scores were distributed from 4 to 33 points (average 14.5) and 10 to 17 points (average 12.8), respectively. Paired *t* test results showed no significant difference between the 2 technologies regarding the total eye fatigue scores, but there was a significant difference regarding the total posture discomfort scores (Figure 9). In conclusion, posture discomfort can be reduced using the proposed see-through type 3D HMD instead of the traditional eyepieces of the surgical microscope.

## Discussion

### Principal Findings

Surgical microscopy is an indispensable system for microsurgery. Although with currently available systems, surgeons can match their eye-hand coordination during surgical procedures, their eyes must be kept on the fixed eyepieces of the microscope for long periods, which requires them to maintain uncomfortable postures that can cause a musculoskeletal disorder. To provide surgeons with the freedom to change their viewing positions, we proposed a high-resolution, see-through type 3D HMD–based surgical microscope system. With this system, surgeons can obtain high-definition 3D surgical images with more comfortable and flexible postures by acquiring the images from a 3D HMD mounted on a headband, instead of keeping their eyes on fixed eyepieces. Furthermore, surgeons can observe a broader surgical environment in addition to the local surgical field using the optical see-through type configuration in the HMD design. Although we used an optical window to block the intensity of the physical view over 90% to provide a brighter surgical image, the see-through type configuration helps in obtaining the peripheral view more easily than a nonsee-through type configuration.

In this proposed system, we used a headband design to remove the weight from the user’s nose. In addition, we used light-weight material for the optical module, and the nonessential area was cut away to minimize the weight of the HMD. However, additional efforts are required to reduce the weight while maintaining a large field of view. Using a thin and light-weight optical module, such as a waveguide or light guide, an HMD with more compact glasses can be achieved [20]. With recent technologies, additional functions of the HMD are easily accessible, such as voice control and a display of additional medical information. Using several HMDs, assistants or students can see the same surgical images as surgeons, which will be helpful in making surgeries and medical education more effective. We are planning to improve the proposed system in our future work.

The results of the feasibility study conducted by preclinical trial surgery showed that the see-through type 3D HMD could provide surgical images with performance similar to that of conventional eyepieces in terms of eye fatigue. In this test, the eye fatigue scores of a few items, including dizziness, were shown to be slightly higher when the HMD was used. One of the main reasons for these increased scores was interpupillary distance (IPD) mismatch. Correspondence between the observer’s IPD and the distance between the optical centers of the binocular HMD is critical for visual comfort. In this work, we set a fixed IPD of 65 mm, a value within the IPD range (50-74 mm) of most adult females and males. Thus, some people who have a short or long IPD can feel dizzy or feel soreness in eyes. Such fatigue can be reduced by adopting adjustable frames of both optical modules for IPD adjustment. We plan to apply an IPD controllable frame in the next version of the HMD. In addition, horizontal, vertical, or rotational deviations of the input images to the eye can bring about eye fatigue or dizziness. Therefore, the alignment of the 2 cameras of the microscope and the 2 microdisplays is also important for reducing eye fatigue. In this work, alignment correction is included in the image processing software for the 2 camera alignments. In addition, fine alignment of the 2 microdisplays has been performed during the packaging process of the optical module.

### Conclusions

In this study, we have introduced a high-resolution, see-through type 3D HMD–based surgical microscope system that is suitable for stable and comfortable microsurgery with advanced functions, such as flexibility of viewing posture and a broad surgical view. The results of the preclinical examination showed that the proposed system has sufficient performance to relieve posture discomfort. The proposed system would be advantageous in overall ENT surgery. To be specific, the main application fields are mastoidectomy and tympanoplasty because they both require long surgical times.

## Abbreviations

CCD: charge-coupled device
ENT: ear, nose, and throat
HDMI: high-definition multimedia interface
HMD: head-mounted display
IPD: interpupillary distance
